# Predictors of adverse pathologic features after radical prostatectomy in low-risk prostate cancer

**DOI:** 10.1186/s12885-018-4416-4

**Published:** 2018-05-09

**Authors:** Jae Won Park, Dong Hoon Koh, Won Sik Jang, Kang Su Cho, Won Sik Ham, Koon Ho Rha, Sung Joon Hong, Young Deuk Choi

**Affiliations:** Department of Urology, Urological Science Institute, Yonsei University College of Medicine, 50-1, Yonsei-ro, Seodaemun-gu, Seoul, 120-752 South Korea

**Keywords:** Low-risk prostate cancer, Adverse pathologic features, Needle biopsy, Radical prostatectomy

## Abstract

**Background:**

Prostate-specific antigen (PSA) screening more frequently detects early stage prostate cancer (PC). However, adverse pathologic features (APFs) after radical prostatectomy (RP) in low-risk PC occur. Previous related studies had utilized outdated staging criteria or small sample cohorts. In this study, we analyzed predictors of APFs after RP in low-risk PC using classification under the current criteria.

**Materials and methods:**

We retrospectively reviewed medical records of 546 low-risk PC patients who had undergone RP. Low-risk PC was defined as PC with clinical T1–T2a, Gleason score ≤ 6, and PSA levels < 10 ng/mL. Clinical and pathological parameters were analyzed to predict APFs. APFs were defined as extracapsular extension (ECE), seminal vesicle invasion (SVI), or positive surgical margins (PSM). We analyzed our data using univariable and multivariable logistic regression analyses, as well as receiver operator characteristics to predict APFs.

**Results:**

Among 546 patients, ECE, SVI, and PSM were present in 199 (36.4%), 8 (1.5%), and 179 cases (32.8%), respectively. PSM had a significant correlation with preoperative high PSA levels and number of positive cores obtained. ECE/SVI was also significantly correlated with PSA levels and number of positive cores. As a result, presence of APFs after RP was associated with high PSA levels and large number of positive cores. PSA > 4.5 ng/mL and number of positive cores > 2 in low-risk PC were significantly associated with APFs, and suggested as cut-off values for predicting APFs.

**Conclusions:**

PSA > 4.5 ng/mL and number of positive cores > 2 in low-risk PC were associated with presence of APFs and patients with such records should be considered carefully to provide active surveillance.

**Electronic supplementary material:**

The online version of this article (10.1186/s12885-018-4416-4) contains supplementary material, which is available to authorized users.

## Background

Prostate cancer (PC) is the most common type of newly diagnosed malignancy in males [1] and it accounts for nearly 30% of all diagnosed male cancers [2]. As PC screening by measuring prostate-specific antigen (PSA) levels has become more widespread, the proportion of PC presenting with low-risk factors has also increased. Therefore, ways to manage this disease have changed significantly [3]. Although initial therapy for PC is determined by risk classification, all treatment options can have negative impacts on the patient’s quality of life [4]. Over-diagnosing and over-treating have thus become major concerns for urologists, especially in regards to low-risk PC [5].

Radical prostatectomy (RP) is a commonly accepted treatment option; however, the possibility of complications during surgical process is a considerable risk factor. Although introduction of robotic surgery has provided significant improvements in outcomes, the risks for urinary incontinence and sexual dysfunction still exist [6, 7]. For now, active surveillance (AS) is being considered for select patients with low-risk, organ-confined PC [2]. Several standards have also been proposed to assess the utility of AS [8]. However, in some studies, patients with low-risk PC, who were eligible for AS, showed adverse pathologic features (APFs) after RP [9, 10]. Despite stringent selection of only low-risk PC, only a quarter of AS patients had pathologically insignificant PC [10]. Even in the low-risk PC group, PC patients with APFs after RP should be considered for adjuvant radiotherapy [11] and these patients should avoid under-treatment. There have been several reports which propose criteria to predict worse pathologic outcome; however, predictors for APFs in low-risk PC group by current criteria are limited. In this study, we aimed to investigate predictors for APFs following RP in the low-risk PC group.

## Methods

Data were collected after approval from the Institutional Review Board at Yonsei University College of Medicine (No. 4–2017-0492). Authors retrospectively reviewed medical records of 4440 patients who underwent RP from 1992 to 2014. RP was performed by multiple surgeons using open or robot-assisted laparoscopic techniques. After exclusion of patients with incomplete medical records and those who received neoadjuvant therapy, patients were classified for preoperative risk group according to NCCN® guidelines. Low-risk PC was defined as PC with clinical T1–T2a, Gleason score (GS) ≤ 6, and PSA levels < 10 ng/mL. Grading system was used according to the 2005 International Society of Urological Pathology (ISUP) Consensus [12]. TNM stage was determined according to the American Joint Committee on Cancer’s 8th edition TNM staging system.

According to our selection criteria, 546 patients with low-risk PC were included in this study. Data for these patients included age, body mass index (BMI), type of operation, preoperative PSA, prostate volume measured by transrectal ultrasonography (TRUS), number of prostate biopsy cores taken, number of positive cores observed, percentage of positive cores, and pathologic characteristics of specimens following RP. Maximal percentage in each biopsy core was defined as the highest percentage of tumor present in each individual biopsy core. All pathologic diagnosis was performed by expert pathologists. Biopsy specimens obtained from outside of our hospital were reviewed by our pathologists. The primary endpoint was occurrence of APFs after RP in low-risk PC group. APFs were defined as extracapsular extension (ECE), seminal vesicle invasion (SVI), and positive surgical margin (PSM) [13]. Tumor volume, which was defined as the combined volume of all nodules, was calculated using a grid method [14].

Univariable and multivariable logistic regression analyses were performed on clinical parameters to investigate predictors for APFs following RP in our low-risk PC cohort. In addition, clinical predictors for each type of APF were also analyzed. Receiver operator characteristics (ROC) curve analysis was performed to determine optimal cut-off value via area under curve (AUC). Youden’s Index in ROC curves was used to select an optimal cutoff value of related parameters for predicting APFs. All statistical analysis was performed out using SPSS Statistics software version 23.0 (IBM, Armonk, NY, USA).

## Results

Baseline characteristics of patients in the study group are displayed in Table 1. Median age for patients was 64 years (Interquartile range [IQR] 59–69). Median prostate volume, as measured by TRUS, was 24.1 ml (IQR 25–42), and median PSA level was 5.6 ng/mL (IQR 4.5–7.0). Median number of biopsy cores taken was 12 (IQR 12–12) with a median number of two positive cores (IQR 1–3). Median percentage of positive cores was 16.7% (IQR 8.3–25.0). Additionally, median maximal percentage in each positive core was 30.0% (IQR 10.0–50.0). Median tumor volume of specimens following RP was 0.8 ml (IQR 0.3–1.8). ECE was present in 199 cases (36.4%), and surgical margins were involved in 179 cases (32.8%). Invaded seminal vesicles were observed in eight cases (1.5%). No lymph node metastasis was reported. GS was upgraded in 210 cases (38.5%). Among them, upgrading to GS 7 (3 + 4) was most common upgrade (68.1%). Furthermore, GS above 8 was also reported in 19 cases (3.5%). Perineural invasion was reported in 175 cases (32.1%) and lymphovascular invasion was present in 12 cases (2.2%). PC with high grade prostatic intraepithelial neoplasia was observed in 308 cases (56.4%).

**Table 1:** Baseline characteristics

Variables (*n* = 546)	Median	IQR
Age, year	64	59–69
BMI, kg/m2	24.1	22.3–25.7
Year of operation (n/%)		
~ 2005	46	8.4
2006~ 2009	365	66.8
2010~ 2014	135	24.7
Type of operation (n/%)		
Open	146	26.7
Robotic	400	73.3
Prostate volume measured by TRUS, ml	31.0	25.0–42.0
PSA, ng/ml	5.6	4.5–7.0
Number of biopsy core	12	12–12
Number of positive core	2	1–3
Percentage of positive core, %	16.7	8.3–25.0
Maximal percentage in each biopsy core, %	30.0	10.0–50.0
Prostate volume in specimen, ml	33.0	26–41.1
Tumor volume in specimen, ml	0.8	0.3–1.8
Tumor / Prostate ratio	0.03	0.01–0.06
Extracapsular extension (n/%)	199	36.4
Seminal vesicle invasion (n/%)	8	1.5
Positive surgical margin (n/%)	179	32.8
GS after radical prostatectomy (n/%)		
GS 6 (3 + 3)	336	61.5
GS 7 (3 + 4)	143	26.2
GS 7 (4 + 3)	48	8.8
GS 8 (4 + 4)	13	2.4
GS 9 or 10	6	1.1
GS upgrading (n/%)	210	38.5
Perineural invasion (n/%)	175	32.1
Lymphovascular invasion (n/%)	12	2.2
HGPIN (n/%)	308	56.4

BMI=Body mass index; GS = Gleason score; HGPIN=High grade prostatic intraepithelial neoplasia; PSA = Prostate specific antigen; TRUS = Transrectal ultrasonography

Univariable and multivariable logistic regression analyses were performed with each clinical parameter for APFs and each component of APFs. While performing univariable and multivariable logistic regression analyses for PSM and PSA levels, number of positive cores was included as covariates in multivariable logistic regression model. A positive association was determined by PSA levels (hazard ratio [HR] 1.14, 95% confidence interval [CI] 1.03–1.25) and number of positive cores (HR 1.12, 95% CI 1.01–1.24), as reported in Table 2.

**Table 2:** Univariable and Multivariable analysis of factors associated with PSM

	Univariable		Multivariable	
	HR (95% CI)	*p* Value	HR (95% CI)	*p* Value
Age	1.01 (0.98–1.03)	0.64		
BMI	1.02 (0.95–1.09)	0.58		
Type of operation				
Open	1 (ref)			
Robotic	1.35 (0.89–2.04)	0.16		
Prostate volume measured by TRUS	1.00 (0.99–1.02)	0.53		
PSA	1.15 (1.05–1.27)	< 0.01	1.14 (1.03–1.25)	0.01
Number of positive biopsy core	1.14 (1.03–1.26)	0.01	1.12 (1.01–1.24)	0.03
Percentage of positive biopsy core	1.01 (1.00–1.02)	0.05		
Maximal percentage in each positive core	1.00 (0.99–1.01)	0.57		

BMI=Body mass index; PSA = Prostate specific antigen; PSM = Positive surgical margin; TRUS = Transrectal ultrasonography

According to the logistic regression analysis which included PSA level, number and percentage of positive cores and maximal percentage in each positive cores as covariates, ECE/SVI was also significantly associated with PSA levels (HR 1.21, 95% CI 1.09–1.33) and number of positive cores (HR 1.38, 95% CI 1.23–1.56), as reported in Table 3. Age, BMI, type of operation, and prostate volumes measured by TRUS were not associated with adverse outcomes.

**Table 3:** Univariable and Multivariable analysis of factors associated with ECE/SVI

	Univariable		Multivariable	
	HR (95% CI)	*p* Value	HR (95% CI)	*p* Value
Age	1.00 (0.98–1.03)	0.77		
BMI	1.03 (0.96–1.10)	0.40		
Type of operation				
Open	1(ref)			
Robotic	1.47 (0.98–2.20)	0.07		
Prostate volume measured by TRUS	0.99 (0.98–1.00)	0.21		
PSA	1.24 (1.12–1.36)	< 0.01	1.21 (1.10–1.33)	< 0.01
Number of positive biopsy core	1.40 (1.25–1.58)	< 0.01	1.38 (1.23–1.56)	< 0.01
Percentage of positive biopsy core	1.04 (1.02–1.05)	< 0.01	1.01 (0.98–1.04)	0.48
Maximal percentage in each positive core	1.02 (1.01–1.02)	< 0.01	1.01 (1.00–1.02)	0.13

BMI=Body mass index; ECE = Extracapsular extension; PSA = Prostate specific antigen; SVI=Seminal vesicle invasion; TRUS = Transrectal ultrasonography;

PSA levels showed a significant correlation with presence of APFs (HR 1.21, 95% CI 1.10–1.33) when analyzed for integrated APFs by same regression analysis model, and number of positive cores was also correlated with worse pathologic outcomes (HR 1.33, 95% CI 1.17–1.50), as reported in Table 4.

**Table 4:** Univariable and Multivariable analysis of factors associated with APFs

	Univariable		Multivariable	
	HR (95% CI)	*p* Value	HR (95% CI)	*p* Value
Age	1.01 (0.99–1.03)	0.40		
BMI	1.05 (0.98–1.12)	0.17		
Type of operation				
Open	1(ref)			
Robotic	1.36 (0.93–2.00)	0.11		
Prostate volume measured by TRUS	1.00 (0.99–1.02)	0.44		
PSA	1.23 (1.13–1.35)	< 0.01	1.21 (1.10–1.33)	< 0.01
Number of positive biopsy core	1.35 (1.19–1.52)	< 0.01	1.33 (1.17–1.50)	< 0.01
Percentage of positive biopsy core	1.03 (1.02–1.05)	< 0.01	1.01 (1.98–1.04)	0.43
Maximal percentage in each positive core	1.01 (1.00–1.02)	< 0.01	1.01 (1.00–1.01)	0.29

**Fig. 1:**
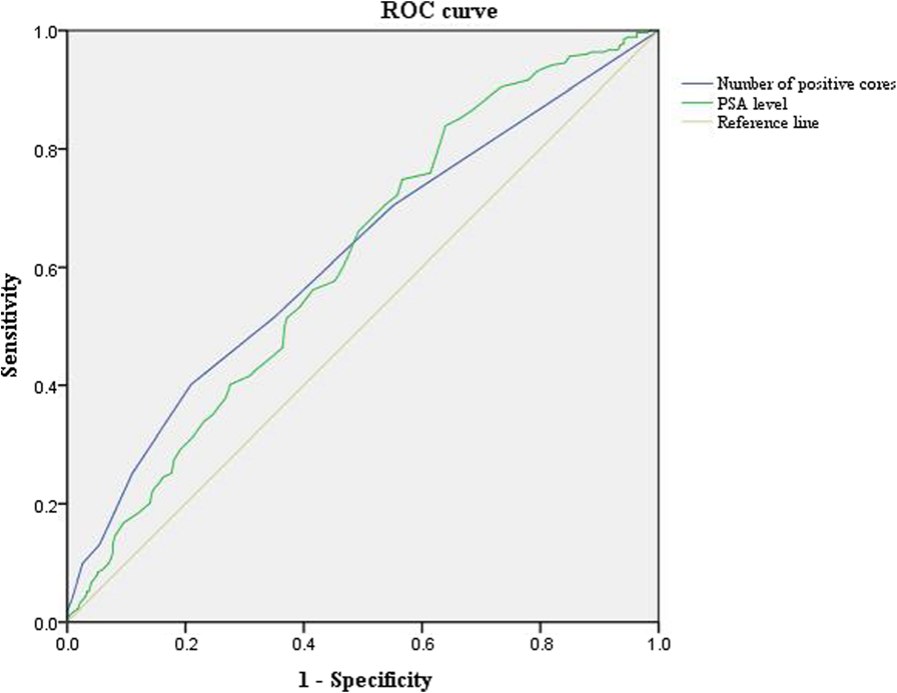
Receiver operator characteristics (ROC) curve of PSA levels and number of positive cores for predicting presence of adverse pathologic features

APFs = Adverse pathologic features; BMI=Body mass index; PSA = Prostate specific antigen; TRUS = Transrectal ultrasonography;

ROC curve analysis was used to determine optimal cut-off value by Youden Index in Fig. 1. Optimal cut-off value for PSA levels, which can predict APFs, was determined to be 4.5 ng/mL, with AUC of 0.61 (95% CI 0.56–0.66). We determined the optimal cutoff value for number of positive cores above 2, which estimated an AUC of 0.62 (95% CI 0.57–0.66). If we considered both parameters to predict APFs, it would have shown slightly better outcomes for predictions in Additional file 1: Figure S1. (AUC = 0.66).

## Discussion

PC has been known as one of the most common cancer types in newly diagnosed population, and the third-leading cause of death in males [1]. Proportion of low-risk PC has increased, and treatment options for PC are known to differ by risk classification [3]. However, substantial variation in treatment exists in the management of localized PC. Localized PC has been treated by not only RP, but also by external beam radiation and AS [15]. Due to concerns of over-diagnosis and over-treatment, AS is considered especially in cases of low-risk localized PC [16]. AS has become one of the treatment options for low-risk PC, under the current NCCN® guidelines [11].

However, in previous studies, APFs were noted after RP even in low-risk PC cases [10, 17]. Beauval et al. reported that only 26% of patients had “insignificant” PC after RP, out of 919 total patients eligible for AS. They reported that 12.5% of patients had extraprostatic extensions, and GS was upgraded in 34% of patients [10]. Simon et al. investigated pathologic outcomes of 1097 PCs in candidates for AS. Of these patients, 7% to 19% had ECE, and 2% to 9% had SVI [17]. Having PSM was also associated with poor prognosis when analyzed for recurrence-free survival and the need for salvage therapy. Ploussard et al. reported that five-year recurrence-free survival occurred in 57.5% of cases with PSM, compared to 84.4% of cases with negative margins [18]. Therefore, prediction of APFs is important in low-risk PC patients who are eligible for AS. In our study, approximately 36% of patients had tumor extension through prostate capsule, and 32% of patients reported PSM (Table 1).

Patients with APFs should be considered for adjuvant radiotherapy after RP [13]. Bolla et al. studied long term results in a randomized controlled trial for postoperative irradiation after RP in 1005 high-risk PC patients. They observed biochemical recurrence (BCR) in 39.4% of patients who received adjuvant therapy, compared to 61.8% in the observation group. Patients who underwent postoperative irradiation showed improvements in biochemical progression-free survival rates and local tumor control (EORTC trial 22,911) [19]. Additionally, Swanson et al. evaluated a cohort of 719 patients for pathologic findings and risk of failure. They suggested that patients with any of the following factors were candidates for adjuvant therapy: ECE, SVI, or margin involvement [20]. Therefore, physicians should offer adjuvant therapy to patients with APFs observed at RP [13]. Patients with APFs should consult with both urologist and radiation oncologist for more complete treatment information [21].

Based on these findings, many researchers have investigated predictors for APFs necessitating adjuvant therapy in low-risk PC patients. Although some recent studies discovered new biomarkers for predicting PC aggressiveness [22, 23], these biomarkers have not been widely used in clinical practice yet.

Prostate biopsy yields information on not only the presence of cancer, but also GS, histological subtype, and additional clinical parameters, including number and percentage of positive cores. In a previous study, Gao et al. investigated pathologic outcomes of 62 low-risk PC patients with PSA levels ≤10 ng/mL, biopsy GS ≤ 7, and clinical cT1c–T2b. They suggested that number of positive biopsy cores was associated with PSM [24]. Ogawa et al. investigated predictors for organ confined PC in 54 patients who underwent RP for T1c PC and reported that PSA levels and number of positive cores were independent predictors for organ confined tumor [25]. However, previous studies included a relatively small number of patients, which could cause limitation in predicting APFs. Our results support these findings by demonstrating a significant association between number of positive cores to PSM and pathologic upstaging (Tables 2 and 3). Number of positive cores is known to be associated not only with PSM or upstaging, but also with GS upgrading following RP. Hong et al. investigated 203 patients with low-risk PC defined by D’Amico classification and 81 patients were upgraded to GS ≥7 after RP. They reported preoperative PSA levels and number of positive cores were independent predictors of GS score upgrading after RP [26]. However, they included only upgrading of GS for their results, and not for predictors of upstaging or PSM.

Our findings are similar to those of previous studies which showed associations between APFs and number of positive cores observed. However, the criterion for classification of low-risk PC has become stricter after these studies were published. Current criteria include not only preoperative PSA levels, but also biopsy GS and clinical T stage [11, 27]. Furthermore, low-risk PC is stratified as low-risk and very low-risk PC, based on these clinical variables [2, 11]. Therefore, previous studies utilized outdated staging criteria, in addition to having relatively small sample cohorts and had limited clinical information to suggest cutoff values for predicting APFs in low-risk PC.

We included patients over a wide range of period from 1992 to 2014. However, most of the patients (91.5%) had undergone RP within 2006 to 2014. Therefore, we used the 2005 ISUP grading system for PC. Due to limitations of pathologic reviews of old specimen slides, we could not re-review the slides before 2005. ISUP Consensus was newly introduced in 2014 [28], and further validations will be followed according to the new grading system. Meanwhile, predictive performance of ROC curve was relatively weak in our study, which may be due to limitations of having subjects with PSA < 10 ng/ml or limited number of biopsy cores taken.

Our study had several limitations. First, it was retrospective review performed at single institution; therefore, multi-center, prospective studies are still needed. Second, biopsy protocol was not standardized as there were many patients who did not undergo prostate biopsy at our institution. Though most patients had 12 core biopsies, there were some patients with fewer than 12 core biopsies. Third, low-risk PC has been re-classified more specifically, with the very low-risk PC category added in the latest guidelines [11]. However, in this study, very low-risk PC showed no significant difference in reporting APFs after RP, compared to low-risk PC. Last, presence of APFs was the primary endpoint of this study. We did not perform an analysis for long term survival of these patients. Long term data should be followed to confirm the lasting impact of APFs on low-risk PC.

Despite these limitations, our study remains informative for clinicians who treat patients with low-risk PC. For now, clinical APF predictors that recommended adjuvant therapy are lacking, especially in low-risk PC, as classified by current criteria. This is the largest study to date to investigate predictors for APFs in low-risk PC, as defined by current staging criteria, considering how using both the number of positive cores and PSA levels better predict APFs than by PSA levels alone. In addition, this study determined the optimal cut-off value to predict APFs in preoperative clinical practice. These parameters could predict APFs in low-risk PC, which is classified by factors such as low PSA, GS, and T stages, without additional cost, all of which may serve as essential information before surgical treatment. These findings offer supplemental information to avoid under-treatment of patients. Randomized studies are needed to further confirm our findings. We also anticipate more precise diagnostic tools to become available through gene analysis in the near future, to further accurately diagnose and treat PC.

## Conclusions

PSA > 4.5 ng/mL and number of positive cores > 2 in low-risk PC was associated presence of APFs and these patients should be considered carefully to provide active surveillance. Physicians should be aware of these parameters, which can predict APFs, and should avoid under-treatment of these patients.

## Additional file

Additional file 1: **Figure S1.** Receiver operator characteristics (ROC) curve of PSA levels, number of positive cores and multivariable logistic regression model incorporating PSA levels and number of positive cores for predicting presence of adverse pathologic features. If we considered both parameters to predict APFs, it would have shown slightly better outcomes for predictions (AUC = 0.662). (JPG 31 kb)

## Abbreviations

APFs: adverse pathologic features
AS: active surveillance
AUC: area under curve
BMI: body mass index
ECE: extracapsular extension
GS: Gleason score
PC: prostate cancer
PSA: prostate-specific antigen
PSM: positive surgical margins
ROC: receiver operator characteristics
RP: radical prostatectomy
SVI: seminal vesicle invasion
TRUS: transrectal ultrasonography
