# Supplementary figures and images for: Predictors of adverse pathologic features after radical prostatectomy in low-risk prostate cancer
Source: BMC Cancer. 2018 May 9;18:545. doi: 10.1186/s12885-018-4416-4 (PMC5944136; doi:10.1186/s12885-018-4416-4)

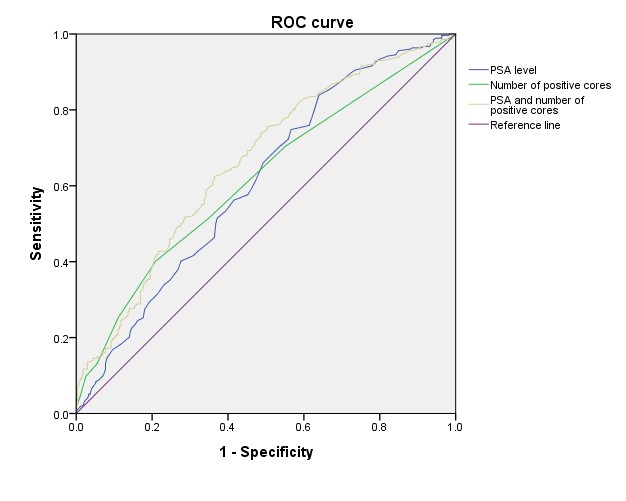

Supplement: Supplementary file 1 — Figure S1. Receiver operator characteristics (ROC) curve of PSA levels, number of positive cores and multivariable logistic regression model incorporating PSA levels and number of positive cores for predicting presence of adverse pathologic features. If we considered both parameters to predict APFs, it would have shown slightly better outcomes for predictions (AUC = 0.662). (JPG 31 kb) [file 12885_2018_4416_MOESM1_ESM.jpg]
